# Finding common connections

**DOI:** 10.7554/eLife.89468

**Published:** 2023-06-26

**Authors:** Ma Francesca M Santiago, Aura Raulo

**Affiliations:** 1 Department of Biology, University of Oxford Oxford United Kingdom; 2 Department of Computing, University of Turku Turku Finland

**Keywords:** gut microbiota, microbiome, correlations between bacteria, universality, personalization, longitudinal data analysis, *P. cynocephalus*

## Abstract

Ecological associations among gut bacteria are largely consistent across hosts in a population of wild baboons.

**Related research article** Roche KE, Bjork JR, Dasari MR, Grieneisen L, Jansen D, Gould TJ, Gesquiere LR, Barreiro LB, Alberts SC, Blekhman R, Gilbert JA, Tung J, Mukherjee S, Archie EA. 2023. Universal gut microbial relationships in the gut microbiome of wild baboons. *eLife* **12**:e83152. doi: 10.7554/eLife.83152.

Living inside the gut of most mammals, including humans, is a unique cocktail of bacteria and other microbes collectively known as the gut microbiome. How individuals end up hosting such varied populations depends not only on the microbes they acquire from their environment, but also on how these organisms ecologically interact with one another inside the gut. For example, some microbes may be excluded from a host by a competitor (negative association; Mäklin et al., 2022), while others may be promoted to grow due to the mutual benefits provided by another microbe (positive association; Seth and Taga, 2014). Which bacteria end up in the microbiome has consequences for the health of the host (Suzuki, 2017). Therefore, understanding how bacteria in the gut interact, and whether these interactions are consistent across individuals, could help researchers develop better therapeutic drugs for recovering and maintaining a healthy gut microbiome.

Negative and positive interactions are typically measured by growing closely related groups of bacteria, known as taxa, in the presence or absence of other taxa (Foster and Bell, 2012; Gould et al., 2018). However, these kinds of experiments are practically impossible to carry out for communities as diverse as the gut microbiome. To overcome this, scientists study individual microbiomes to determine which bacterial taxa frequently occur together (suggesting they promote each other’s growth), and which rarely inhabit the same host (suggesting they are likely to be competitors). However, this approach does not reveal if interactions are consistent across different hosts (i.e. universal), or are unique to bacterial communities inside each individual.

Now, in eLife, Elizabeth Archie from the University of Notre Dame and colleagues – including Kimberley Roche as first author – report how different bacterial taxa fluctuate over time in the gut of wild baboons to determine if the interactions between them are universal (Roche et al., 2023). The team (who are based at various institutes in the United States and Germany) used data from a long-term study of wild baboons living in Amboseli, Kenya which had their microbiomes sampled hundreds of times between 2000 and 2013. Using a statistical model they had developed, Roche et al. tracked how the quantity of individual taxa rose and declined over time. These traces were then compared to determine which bacterial taxa fluctuated together (positive association) and which rose and fell in opposing directions (negative association).

Next, Roche et al. examined if the associations among bacteria were comparable across individuals. The strength and direction of the correlations (i.e. whether they were positive or negative) were remarkably consistent across the 56 baboons studied, suggesting that ecological interactions in the gut microbiome are mostly universal (Figure 1). Most of these associations were relatively weak and negative, suggesting that gut bacteria usually do not help one another grow, but rather compete with each other or thrive in different states, such as hosts with specific variations in their immune or endocrine systems. This result aligns well with ecological theory which predicts that strong, positive dependencies within a community should be rare, as highly interdependent ecosystems are likely to be unstable (Coyte et al., 2015) – a phenomenon Roche et al. refer to as an ‘ecological house of cards’.

**Figure 1.**
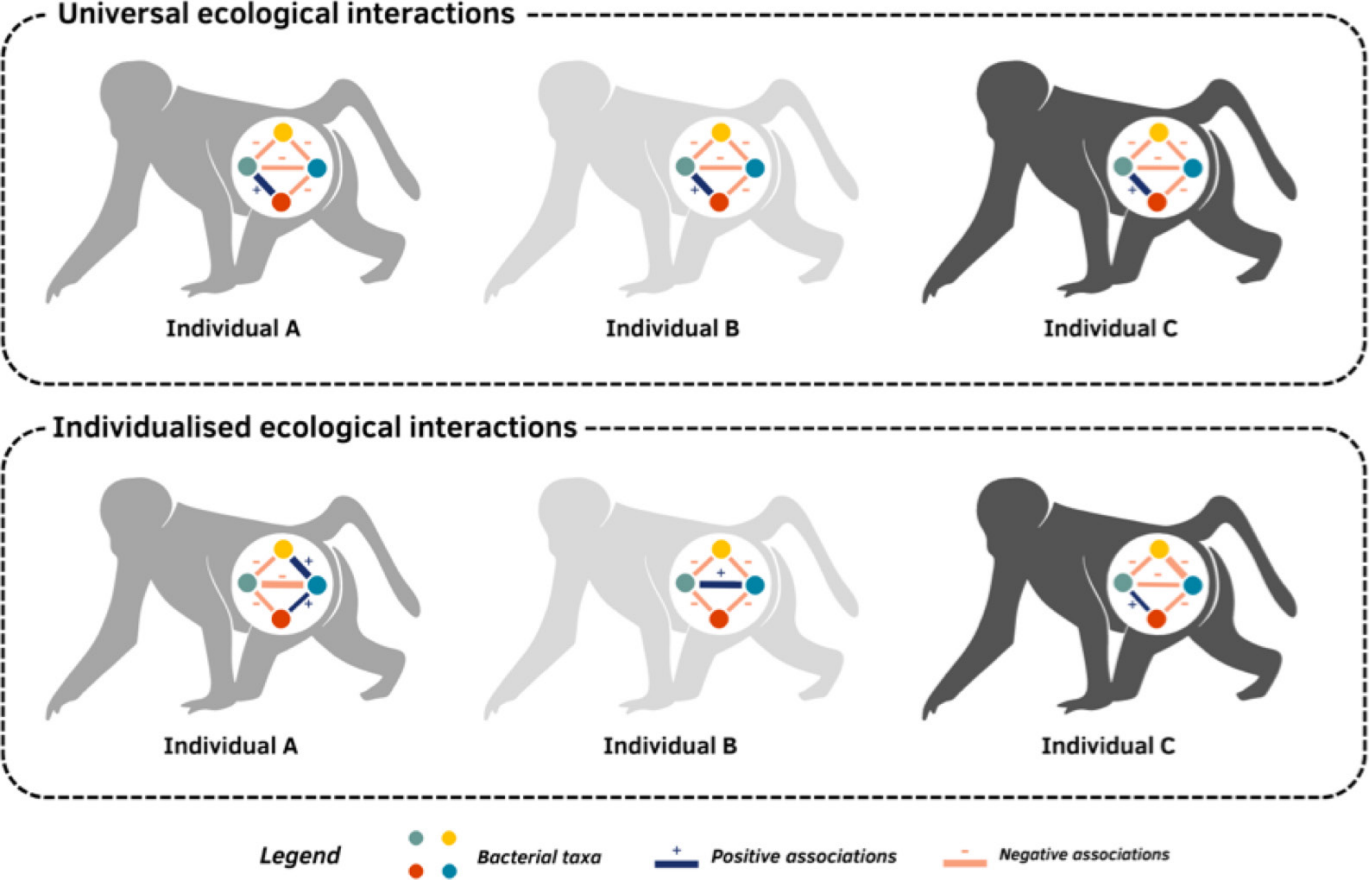
Are interactions between gut bacteria universal or individual to each host?. The community of microbes living in the gut of mammals, such as baboons, varies between individuals. This variation depends on how bacteria from different taxa (represented by coloured circles) interact with one another: some taxa may support each other’s growth (positive association; blue line), while others may compete to prevent one another from inhabiting the same host (negative association; orange line). The strength of these connections can also vary (represented by the width of the line). Roche et al. used data from the gut microbiomes of baboons to investigate if these associations are universal, meaning taxa interact the same way across multiple hosts (top panel), or individualised, meaning taxa have different relationships with one another in different hosts (bottom panel).

It is unclear how many of these universal associations arise from direct ecological interactions between microbes, and how many arise from bacterial taxa simply preferring similar or different host environments. Roche et al. make a compelling case that both of these processes might be at play. They show that environmental similarity (e.g. diet) between hosts does not influence universality scores, supporting the idea that these associations reflect true ecological interactions. Furthermore, they show that associations with the highest universality score were positive relationships between closely related bacteria, suggesting that these interactions may be partly due to bacteria responding to a host’s internal state or environment in a similar way.

These findings suggest that while the composition of microbes in the gut can be highly individualised, the underlying ecology shaping this variation may be similar. This is good news for scientists developing therapies to modify the gut microbiome, such as probiotics, since it means that different microbiomes are likely to react predictably to interventions. Moreover, the ability to infer ecological phenomena from microbiome data has tremendous value for ecology as a whole.

The last century has seen a vast number of mathematical models for describing ecological theories, such as the theory of ecological succession (Cowles, 1899). However, testing these models in large-scale ecosystems is challenging as it usually requires data that has been collected over long periods of time, such as seeing a forest grow from its initial state to a stable climax. The findings of Roche et al. suggest that many hypotheses of ecological theory could instead be tested with data from microbiomes – which vary greatly across space, change quickly, but follow the same ecological principles as the large-scale ecological world we perceive. This new approach could bring scientists one step closer to answering many unexplored questions in ecology which, ultimately, govern our ability to survive on this planet.
